# Rapid improvement in a recurrent generalized pustular psoriasis patient ineffective to Ixekizumab following Spesolimab therapy^☆^

**DOI:** 10.1016/j.abd.2024.09.003

**Published:** 2024-12-30

**Authors:** Wenjie Li, Fan Cui, Lixia Zhang, Ge Yang, Zhen Rang, Minyan Xu, Yangying Liu, Siyu Wang

**Affiliations:** a School of Medicine, University of Electronic Science and Technology of China, Chengdu, China; b Institute of Dermatology, Sichuan Academy of Medical Sciences and Sichuan Provincial People’s Hospital, Chengdu, China; c Department of Pathology, Sichuan Academy of Medical Sciences and Sichuan Provincial People’s Hospital, Chengdu, China

*Dear Editor,*

Generalized pustular psoriasis (GPP) is a rare and dangerous subtype of psoriasis characterized by recurrent episodes of widespread erythema with numerous sterile pustules, often accompanied by varying degrees of systemic inflammatory manifestations.

A 52-year-old female presented with recurrent erythema and pustules accompanied by pain for 2 years, exacerbated after discontinuing oral medications for the past 2 days, along with generalized fatigue, then she was admitted to our department for inpatient treatment. Prior to admission, she relied on long-term oral acitretin (10‒20 mg once daily), prednisone acetate tablets (10 mg three times daily) to control her condition, and received injections of ixekizumab (anti-IL-17A) (80 mg every 2-weeks) for a total of 12-weeks, but the skin lesions did not completely improve, and the pustules continued to recur. Upon physical examination, her temperature was normal, with swelling of the limbs and diffuse erythema almost covering the entire body, containing densely distributed pinpoint-sized pustules. Local skin temperature was elevated with evident tenderness. Generalized Pustular Psoriasis Physician Global Assessment (GPPGA: 11), Generalized Pustular Psoriasis Area and Severity Index (GPPASI: 38.3). Her white blood cell count (13.52 × 10^9^/L), neutrophil count (11.33 × 10^9^/L), neutrophil percentage (83.8%), and CRP (45.75 mg/L) were elevated upon admission, accompanied by decreased albumin (35.5 g/L). Then we performed a skin biopsy and combined the clinical and pathological findings (Fig. 1 A and B), she was diagnosed with GPP. With the patient's consent, we completed the pre-biologic screening and administered a single dose of 900 mg spesolimab intravenously over 90 minutes, the patient experienced no discomfort during or after the injection process. Within 24 hours of Spesolimab treatment, almost complete clearance of pustules was observed, with the erythema darkening in color and beginning to desquamate (GPPGA: 5, GPPASI: 11.7) (Fig. 2 A and B) Five days later, the patient's generalized erythema lightened with minimal desquamation and no new skin lesions observed. Her blood routine, CRP, and albumin returned to normal levels without any signs of new infection (GPPGA: 2, GPPASI: 5) (Fig. 3A). The patient experienced rapid relief of GPP symptoms post-Spesolimab treatment and was discharged after 5-days of treatment. She continued topical corticosteroid cream at the lesion sites post-discharge and remained free of significant erythema and pustules at the 3-month follow-up (Fig. 3B).

**Figure 1:**
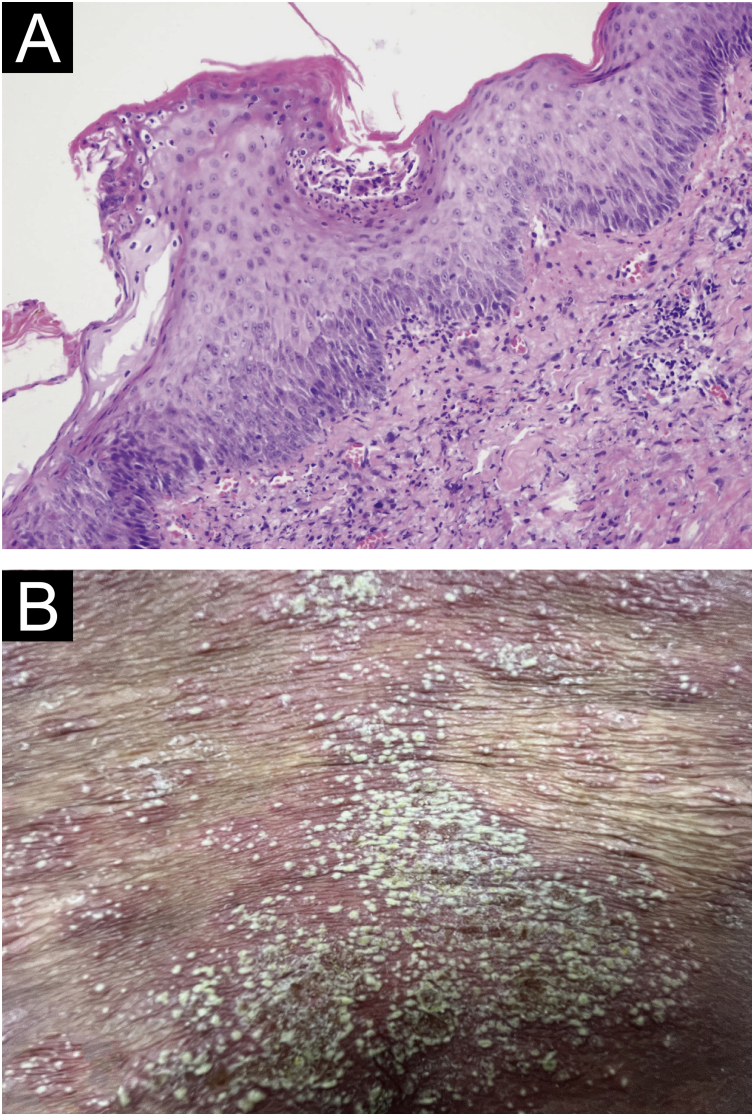
(A) Biopsy results from the skin folds under the breast: Epidermal hyperkeratosis with mild parakeratosis, focal neutrophilic aggregates forming microabscesses in the stratum corneum, thinning of the granular layer, acanthosis in the spinous layer, mild spongiosis in the spinous layer, and dermal perivascular infiltration of inflammatory cells including lymphocytes, histiocytes, and neutrophils (Hematoxylin & eosin, ×200); (B) Localized pustules at admission.

**Figure 2:**
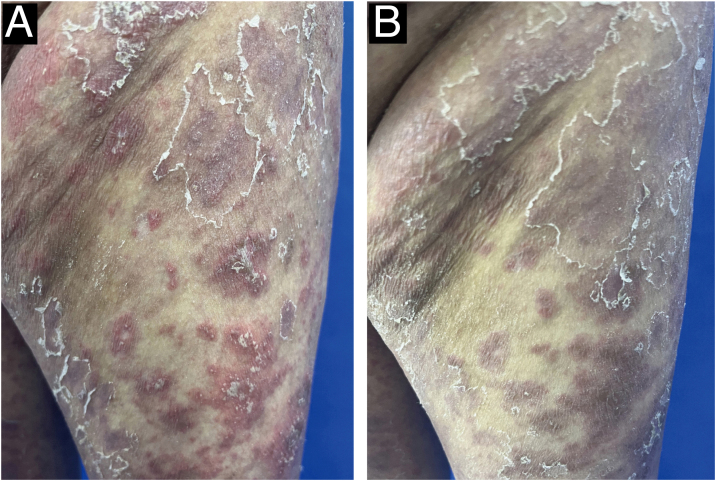
(A) Skin lesions on the left thigh 1-day before Spesolimab treatment; (B) Changes in skin lesions on the left thigh 1 day after Spesolimab treatment.

**Figure 3:**
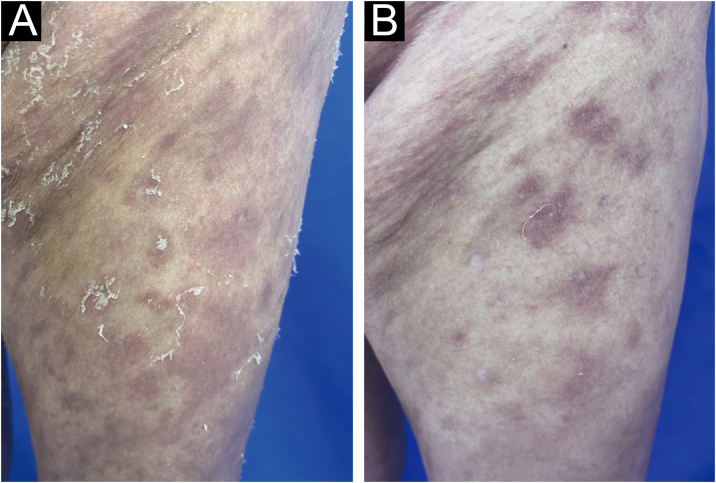
(A) Changes in skin lesions on the left thigh 5-days after Spesolimab treatment; (B) Changes in skin lesions on the left thigh 3-months after Spesolimab treatment.

Due to the rarity and complexity of GPP, there is currently no globally agreed-upon treatment regimen that can completely control GPP flare-ups. Research has revealed a close immunopathological link between GPP and plaque psoriasis, and some biologics used for treating plaque psoriasis, such as anti-TNF-α antibodies, anti-IL-17A antibodies, and anti-IL-12/23 p40 antibodies, have also shown efficacy in treating GPP and have been included in the recommended treatment guidelines for GPP in Japan.^1^ However, studies on their efficacy primarily involve small-scale clinical cases, with limited systematic clinical research or randomized controlled trials, resulting in a lower level of evidence. Although patients with GPP often exhibit clinical manifestations similar to plaque psoriasis, growing evidence suggests that GPP, as a separate clinical entity, is an autoinflammatory pustular neutrophilic disease. Unlike plaque psoriasis, the core of GPP development lies in the IL-1 and IL-36 pathways.^2^ Our case seems to confirm this distinction. Spesolimab, an IL-36 receptor inhibitor, has demonstrated efficacy and safety in rapidly controlling acute GPP episodes through Phase II clinical trials,^3^ making it a promising therapeutic option for GPP management. Additionally, although some evidence suggests that other biologics typically used for plaque psoriasis might also be effective for GPP, several reported cases of GPP had previously attempted treatment with biologics such as ustekinumab, adalimumab, infliximab, and risankizumab (Table 1),^4^, ^5^, ^6^ but the potential efficacy of these biologics in managing GPP was not well demonstrated in these patients. In contrast, these cases achieved rapid and significant relief of skin lesions after spesolimab treatment. Although IL-36 cytokines can induce the expression and interaction of various downstream cytokines like IL-17A, IL-23, and TNF-α, which may also play roles in the pathogenesis of GPP, the role of IL-36 in the clinical treatment targets of GPP may be more crucial and significant. In summary, spesolimab represents a more precise and potent option when other biologics show suboptimal results in treating GPP, and future research may require larger-scale validation to demonstrate its safety and durability.

**Table 1:** Similar cases successfully treated with Spesolimab.

Authors/Year	Patient characteristics and GPP history	Plaque psoriasis history	Initial biologic agent used	Preliminary outcome	Dosage of Spesolimab used	Final outcome
Jiang et al.^4^ 2023	34-years-old, female, 2-years	20-years	Ustekinumab (anti-IL-12/23) 45 mg once; Adalimumab (anti-TNF-α) 80 mg twice	The condition tended to stabilize within 1-week, but new pustules appeared later	A single dose of 900 mg	Complete resolution of skin lesions within 1-week, with no recurrence or adverse reactions during the 5-month follow-up after treatment
Ran et al.^5^ 2023	26-years-old, female, 14-years	Negative	Adalimumab (anti-TNF-α)	Ineffective	A single dose of 900 mg	Complete resolution of skin lesions at 16-weeks post-treatment
Müller et al.^6^ 2023	63-years-old, male, about 4-years	Negative	Infliximab (anti-TNF-α) 5 mg/kg; Risankizumab (anti-IL-23)	Regular recurrences	Two doses of 900 mg	Complete disappearance of pustules, significant reduction in erythema, no relapse
Our case	52-years-old, female, 2-years	Negative	Ixekizumab (anti-IL-17A) initially 160 mg, followed by 80 mg every 2-weeks for a total duration of 12-weeks	Ineffective	A single dose of 900 mg	Complete disappearance of pustules within 24-hs, a significant reduction in erythema, no relapse after 3-months

## Financial support

None declared.

## Authors’ contributions

Wenjie Li: Writing of the manuscript and critical review of important intellectual content.

Fan Cui: Critical review of the literature.

Lixia Zhang: Intellectual participation in the propaedeutic and therapeutic conduct of the studied case.

Ge Yang: Effective participation in the research guidance.

Zhen Rang: Effective participation in the research guidance.

Minyan Xu: Effective participation in the research guidance.

Yangying Liu: Final approval of the final version of the manuscript.

Siyu Wang: Final approval of the final version of the manuscript.

## Conflicts of interest

None declared.
